# Affibody-Mediated Sequestration of Amyloid β Demonstrates Preventive Efficacy in a Transgenic Alzheimer’s Disease Mouse Model

**DOI:** 10.3389/fnagi.2019.00064

**Published:** 2019-03-22

**Authors:** Allal Boutajangout, Hanna Lindberg, Abdulaziz Awwad, Arun Paul, Rabaa Baitalmal, Ismail Almokyad, Ingmarie Höidén-Guthenberg, Elin Gunneriusson, Fredrik Y. Frejd, Torleif Härd, John Löfblom, Stefan Ståhl, Thomas Wisniewski

**Affiliations:** ^1^Center for Cognitive Neurology, New York University Langone Health, New York, NY, United States; ^2^Department of Neurology, New York University Langone Health, New York, NY, United States; ^3^Department of Psychiatry, New York University Langone Health, New York, NY, United States; ^4^Department of Physiology & Neuroscience, New York University Langone Medical Center, New York, NY, United States; ^5^Department of Protein Science, School of Engineering Sciences in Chemistry, Biotechnology and Health, KTH Royal Institute of Technology, Stockholm, Sweden; ^6^School of Medicine, King Abdulaziz University, Jeddah, Saudi Arabia; ^7^Affibody AB, Solna, Sweden; ^8^Department of Chemistry and Biotechnology, Swedish University of Agricultural Sciences (SLU), Uppsala, Sweden; ^9^Department of Pathology, New York University School of Medicine, New York, NY, United States

**Keywords:** Alzheimer’s disease, affibody molecule, amyloid beta (Aβ), behavior, histology, immunotherapy, transgenic mice

## Abstract

Different strategies for treatment and prevention of Alzheimer’s disease (AD) are currently under investigation, including passive immunization with anti-amyloid β (anti-Aβ) monoclonal antibodies (mAbs). Here, we investigate the therapeutic potential of a novel type of Aβ-targeting agent based on an affibody molecule with fundamentally different properties to mAbs. We generated a therapeutic candidate, denoted Z_SYM73_-albumin-binding domain (ABD; 16.8 kDa), by genetic linkage of the dimeric Z_SYM73_ affibody for sequestering of monomeric Aβ-peptides and an ABD for extension of its *in vivo* half-life. Amyloid precursor protein (APP)/PS1 transgenic AD mice were administered with Z_SYM73_-ABD, followed by behavioral examination and immunohistochemistry. Results demonstrated rescued cognitive functions and significantly lower amyloid burden in the treated animals compared to controls. No toxicological symptoms or immunology-related side-effects were observed. To our knowledge, this is the first reported *in vivo* investigation of a systemically delivered scaffold protein against monomeric Aβ, demonstrating a therapeutic potential for prevention of AD.

## Introduction

Alzheimer’s disease (AD) is the most common cause of dementia and is defined by a gradual onset and progression of deficits in several areas of cognition. More than 45 million people worldwide are affected and the associated costs on the society are enormous (Wisniewski and Goñi, 2015). Historically, AD has been characterized by its pathological signatures that include e.g., extracellular deposits of amyloid β (Aβ) and intracellular neurofibrillary tangles (NFTs; Nelson et al., 2012). Aβ peptides of various lengths are formed through sequential cleavage of the amyloid precursor protein (APP) by β- and γ-secretases. An important event in AD involves aggregation of soluble monomeric peptides into neurotoxic and insoluble β-sheet-rich inclusions in the brains of patients (Hardy and Selkoe, 2002). Present therapies for AD have either no or minimal disease-modifying effect, and thus, there is an urgent need for new effective treatments. Numerous therapeutic strategies are under investigation to delay the onset or slow progression of the disease (Wisniewski and Goñi, 2015; van Dyck, 2018). Active and passive immunotherapeutic approaches have been suggested to improve clinical progression and cognitive impairment through different mechanisms: (i) inhibition of Aβ production; (ii) interference with the formation of toxic aggregation intermediates; and (iii) accelerated clearance of Aβ from the CNS into the periphery (Citron, 2010; Karran et al., 2011). Several anti-Aβ antibodies have demonstrated effective clearance of Aβ together with cognitive improvements in transgenic animal models (Bard et al., 2000; DeMattos et al., 2001; Wilcock et al., 2004) and consequently progressed to clinical trials (Sevigny et al., 2016; Siemers et al., 2016; Herline et al., 2018b). However, translation to safe and efficacious therapies for humans has been challenging as AD clinical trials have failed to show sufficient clinical benefits (Doody et al., 2014; Salloway et al., 2014; Herline et al., 2018b). Recently, the monoclonal antibody (mAb) Solanezumab, that binds monomeric Aβ, was extensively evaluated in a phase III prevention trial in patients with mild AD. The study was however terminated due to failure in showing cognitive improvements. A recent phase Ib trial with the protofibril-binding mAb Aducanumab has demonstrated promising results, which has motivated an on-going phase III trial (Sevigny et al., 2016).

It has been proposed that challenges related to the failure in showing overall clinical improvement or clear disease-modifying results of these mAbs could be addressed to some of the inherent properties of antibodies (Pul et al., 2011; Herline et al., 2018b). Thus, new approaches based on engineered antibody domains or alternative scaffold-proteins that generally lack immunoglobulin-related effector functions are now investigated and moving into clinical development, as they might provide safer and more effective treatments (Robert and Wark, 2012; Nisbet et al., 2013; Vazquez-Lombardi et al., 2015). Antibody derivatives and non-immunoglobulin affinity proteins are in general smaller than full-length antibodies, and can typically be engineered into multivalent and multispecific constructs with an overall size that is still much smaller than a full-sized antibody (Löfblom et al., 2010; Robert and Wark, 2012; Vazquez-Lombardi et al., 2015; Ståhl et al., 2017). Their smaller size could potentially result in a different *in vivo* biodistribution profile as well as simplified administration routes, which could be important in the treatment of e.g., AD.

Affibody molecules represent a class of promising alternative scaffold proteins that have been investigated for various applications (Ståhl et al., 2017). Affibody molecules are small (6.5 kDa), three-helical bundle proteins, typically with high solubility, high expression yields in bacteria, the possibility of chemical synthesis as an alternative production strategy, and straightforward engineering of bispecific and bivalent constructs (Bass et al., 2017; Ståhl et al., 2017), which is often valuable for development of therapeutic constructs. Affibody molecules have been generated to numerous target proteins, with typical affinities in the low nanomolar to picomolar range (Ståhl et al., 2017). They have demonstrated significant potential as medical imaging agents, and have been generated to several different cancer biomarkers (Ståhl et al., 2017). A human epidermal growth factor receptor 2-targeting affibody molecule has been extensively evaluated in clinical trials as a breast cancer-imaging agent (Sörensen et al., 2014, 2016), which proved to be safe and efficacious.

For therapeutic applications, extended *in vivo* circulation times are generally required. Affibody molecules can be genetically fused to a 46 amino acid (5.2 kDa) albumin-binding domain (ABD), that has been deimmunized (Andersen et al., 2011; Frejd, 2012) and engineered to femtomolar affinity (Jonsson et al., 2008). This concept of half-life extension has been successfully explored in several preclinical affibody-based therapy studies (Tolmachev et al., 2007; Ståhl et al., 2017) and is currently being evaluated in a phase II clinical study where the half-life extension is used in combination with an IL-17 specific affibody for treatment of plaque psoriasis (Ståhl et al., 2017).

We have previously reported on the generation of an affibody molecule (denoted Z_Ab3_) that binds to monomeric Aβ with a 17 nM affinity (Grönwall et al., 2007; Hoyer et al., 2008). This binder was evolved to adopt a unique structure upon binding of monomeric Aβ by engaging two identical disulfide-linked affibody units to sequester the aggregation-prone residues of the peptide in a tunnel-like cavity (Hoyer et al., 2008). Upon binding, both affibody units and the Aβ peptide undergo structural rearrangement and form an internal stabilizing β-sheet conformation (Hoyer and Härd, 2008), which might be more efficient for interactions with aggregation-prone peptides. This Aβ-sequestering affibody molecule has demonstrated efficient inhibition of formation of Aβ aggregates in an *in vivo* *Drosophila* AD model, and abolished the neurotoxic effects as well as restored the life span of the flies (Luheshi et al., 2010). The affibody molecule was further engineered into a truncated genetic dimer, thus reducing the overall size to 11.2 kDa, and increasing the affinity to Aβ (340 pM; Lindberg et al., 2015). In an *in vitro* binding assay, this second-generation Aβ-capturing affibody molecule (denoted Z_SYM73_), genetically linked to an ABD at the C-terminal, demonstrated efficient capture of physiological concentrations of monomeric Aβ from a complex mixture of proteins while simultaneously binding to serum albumin *via* the ABD, an important feature in a potential therapeutic setting (Lindberg et al., 2015). It was speculated that Z_SYM73_ could be an interesting candidate to assess as a prevention drug for AD in relevant animal models (De Genst and Muyldermans, 2015).

Encouraged by these positive results, we here investigate the efficacy of Z_SYM73_-ABD (total size 16.8 kDa) as a therapeutic candidate to prevent the development of AD-related pathology in transgenic AD mice. Z_SYM73_-ABD and a negative control protein (a dimeric variant of a *Taq* polymerase-binding affibody molecule genetically linked to ABD) were produced in *E. coli* and recovered to high purity, and the preventive efficacy was assessed in APP/PS1 double transgenic mice (Holcomb et al., 1998; Puzzo et al., 2015). The animals received three weekly injections of 100 μg therapeutic protein or negative control protein during 13 weeks, starting at the expected onset of pathology development. Extensive behavioral assessment together with histological evaluation demonstrated a significantly lower amyloid burden in both cortex and hippocampus, as well as rescued cognitive functions of the Z_SYM73_-ABD treated mice relative to controls. This study is the first *in vivo* investigation of a systemically delivered scaffold protein binding to monomeric Aβ, which demonstrates a preventive therapeutic efficacy on the development of disease-related pathology in a mouse model of AD.

## Materials and Methods

### Production of Affibody Fusion Proteins

Gene fragments encoding Z_SYM73_ (Lindberg et al., 2015) or a dimer of the control affibody molecule Z_Taq_ (Gunneriusson et al., 1999) were fused to the gene for a deimmunized high-affinity ABD (Jonsson et al., 2008; Frejd, 2012; Malm et al., 2013) and inserted into expression vectors containing a T7 promoter system and a kanamycin resistance gene. The vectors thus encoded the fusion proteins (Z_SYM73_)-GAPG_4_STS-ABD (hereinafter denoted Z_SYM73_-ABD) and (Z_Taq_)_2_-GAPG_4_STS-ABD [hereinafter denoted (Z_Taq_)_2_-ABD]. DNA sequence verification was performed using BigDye Thermo Cycle Sequencing reactions with an ABI Prism 3700 instrument (Applied Biosystems, Foster City, CA, USA). The affibody-fusion proteins were produced in* E. coli* BL21 DE3 cells (Novagen, Madison, WI, USA) with protein expression induced by 0.2 mM isopropyl β-D-1-thiogalactopyranoside (IPTG), followed by cultivation in a multi-fermentor system (GRETA, Belach Bioteknik AB, Solna, Sweden). The cells were harvested, disrupted by sonication, and purified, in principle as described previously (Lindberg et al., 2015) however using an affinity chromatography column with an anti-ABD Sepharose matrix (Affibody AB, Solna, Sweden). Additional purification steps were performed using reverse phase chromatography on an ÄKTA Explorer 100 system, and size exclusion chromatography using a HiLoad 16/60 column together with an ÄKTA system (GE-Healthcare, Uppsala, Sweden) and using PBS as running buffer. After purification, potential residual endotoxins were removed by passing the proteins through 1-mL EndoTrap columns (Hyglos), according to the supplier’s recommendations. The proteins were eluted in DPBS (Gibco, Life Technologies), followed by determination of endotoxin levels (APJ, Stockholm). A hexahistidine-tagged Z_SYM73_ affibody molecule (hereinafter denoted Z_SYM73_-His_6_) was also produced and purified as previously described (Lindberg et al., 2015).

### Characterization of Affibody Proteins

The molecular masses of the proteins were analyzed using MALDI mass spectrometry (4800 MALDI TOF-TOF, Sciex) and SDS-PAGE under both reducing and non-reducing conditions. The secondary structure content was analyzed at a concentration of 0.5 mg/mL in PBS using circular dichroism (CD) spectroscopy on a Chirascan spectropolarimeter (Applied Photophysics, United Kingdom) in a quartz cell with an optical path-length of 1 mm. CD spectra were collected from 250 to 195 nm at 20°C, before and after variable temperature measurement. The thermal stability (variable temperature measurement) was measured at 221 nm while heating the proteins from 20 to 90°C (1°C/min). In-solution kinetics and affinity between Z_SYM73_-ABD and Aβ_40_, Aβ_42_ [both from AnaSpec, San Jose, CA, USA; in the presence of human serum albumin (HSA)] and HSA (Albucult, Sigma-Aldrich), respectively, were determined using a kinetic exclusion assay (KinExA, Sapidyne Instruments Inc., Boise, ID, USA). In addition, the binding ability of Z_SYM73_-His_6_ (Lindberg et al., 2015) to Aβ_40_ and Aβ_42_ (AnaSpec, San Jose, CA, USA), respectively, were measured. The k_a_ and the K_D_ of the different interactions were measured in separate analyses. Briefly, the k_a_ values were determined by incubating constant concentrations of affibody molecule and target protein and measuring the free amount of affibody molecule after different incubation times. The K_D_ values were measured by incubation of varying concentrations (two-fold dilutions) of the target protein with constant concentrations of affibody molecule and measuring the free amount of affibody molecule after a constant incubation time. In both types of measurements, free amount of affibody molecule was quantified using streptavidin-coated PMMA beads (Sapidyne Instruments), immobilized with biotinylated Aβ_40_ or biotinylated HSA depending on the binding event that was measured. Detection was achieved using a mouse anti-His_6_ IgG (Sapidyne Instruments) for Z_SYM73_-His_6_ analyses, a mouse anti-affibody mAb (Affibody AB) for HSA-binding and a mouse anti-HSA mAb (Abcam, Cambridge, UK) for Aβ_40_ and Aβ_42_-binding for Z_SYM73_-ABD analyses. In all cases, a goat anti-mouse polyclonal antibody (pAb) conjugated with Daylight 650 (Sapidyne Instruments) was used as a secondary step in the detection. Serum stability of Z_SYM73_-ABD was investigated by incubation of Z_SYM73_-ABD in a human plasma pool at 37°C during 72 h at a concentration of 10 μg/mL. Retained affinity to Aβ was investigated in a conventional ELISA with biotinylated Aβ_40_ bound to streptavidin in ELISA wells. Two-step dilutions series of Z_SYM73_-ABD incubated in plasma for 28 days, compared to an untreated sample, were detected using an HRP-conjugated mouse anti-Z mAb (Affibody AB) followed by a one-step ultra-tetramethyl benzidine (TMB; Thermo Fisher Scientific, Rockford, IL, USA) and measured at 450 nm.

### AD Mouse Model and Treatment

This study was carried out in accordance with the recommendations of the New York University School of Medicine Institutional Animal Care and Use Committee. The protocol was approved by the New York University School of Medicine Institutional Animal Care and Use Committee. APP/PS1 (APP_K670N/M671L_ and PS1_M146V_ transgenes) have been extensively used as a model of amyloid plaque deposition (Holcomb et al., 1998; Puzzo et al., 2015). The mice were bred at the NYU School of Medicine and were maintained on a 12 h light/dark cycle. Animals were injected intraperitoneally (i.p.) with either the Aβ-binding Z_SYM73_-ABD or the negative control (Z_Taq_)_2_-ABD affibody protein. Twenty mice were divided into two study groups with 10 animals in each that received three weekly injections of 100 μg affibody molecule (corresponding to a plasma concentration of ~1.5 μM that is estimated to fall to 0.2 μM after 3 days) for 13 weeks starting at the age of 4 months. In this model, amyloid plaque pathology starts at about 3 1/2 months of age; hence treatment was initiated at the beginning of pathology development (Holcomb et al., 1998; Drummond and Wisniewski, 2017). During the treatment, the mice were weighed and examined for general health indicators.

### Plasma Levels of Affibody Molecule

The mice were bled before the commencement of the study (T_0_) and periodically throughout the experiment as follows: 24 h after the 39th injection (T_1_), 7 days after the 39th injection (T_2_), and 14 days after the 39th injection (T_3_). Plasma levels of affibody molecule were detected using ELISA. Aβ peptide was coated onto microtiter wells (Immulon 2HB; Thermo Electron Corp., Milford, MA, USA), following detection of affibody molecules in plasma using a goat α-affibody IgG (1:1,000 dilution, ADIDAS, Affibody AB). Bound α-affibody antibodies were detected using biotinylated anti-goat IgG (Amersham Biosciences, Piscataway, NJ, USA), followed by a Streptavidin HRP. TMB (Pierce, Rockford, IL, USA) was used as a color developing substrate and measured at 450 nm.

### Sensorimotor Activity

Prior to cognitive assessment, the locomotor activity and motor coordination of the mice were tested to verify that any treatment-related effects observed in the cognitive tasks could not be explained by differences in sensorimotor abilities. Before testing, the animals were adapted to the room with lights on for 15 min.

For exploratory locomotor assessment, each mouse was habituated in a circular open field chamber (70 × 9 × 70 cm) for 15 min during which they were allowed to explore the environment. Horizontal movements in each dimension of the open field (i.e., x, y, and two z planes) were automatically recorded by a video camera (Hamilton-Kinder Smart-frame Photobeam System) mounted above the chamber. Results were reported as distance traveled (cm), average and maximum travel velocity (cm/s) and mean resting time (s) of the mouse. After each session, the field was cleaned with water and 30% ethanol.

To assess motor behavior in terms of forelimb and hindlimb coordination as well as balance, each animal was placed onto a 3.6 cm diameter rod (Rotarod 7650 accelerating model; Ugo Basile, 114 Biological Research Apparatus, Varese, Italy). First, the mice received two training trials to reach a baseline level of performance, where after they were tested in three additional trials with an initial speed of 1.5 rpm that was gradually increased by 0.5 rpm every 30 s to a maximum speed of 40.0 rpm. In each trial, the animal was tested in three sessions, each separated by 15 min. A soft foam cushion was placed under the rod to prevent injury from falling. To assess the performance, total time on the rod and rotation speed in rounds per minute were recorded when the animal fell onto a soft foam cushion or inverted (by clinging) from the top of the rotating barrel. The rod was cleaned with water and 30% ethanol after each session.

### Radial Arm Maze

Spatial learning (working memory) was assessed using a radial maze with eight 30-cm arms originating from the central space, with a water-well at the end of each arm (Liu et al., 2014). An age-match group of non-transgenic mice was also tested in the radial arm maze. Clear Plexiglas guillotine doors were operated by a remote pulley system, which controlled access to the arms from the central area from where the animals were allowed to enter and exit all arms of the apparatus. Before testing, the mice were deprived of water for 24 h and then their access to water was restricted to 1 h per day for the duration of testing. After 2 days of adaptation, the mice were subjected to testing for nine consecutive days. This relatively long period of adaptation has been found important as these transgenic mice tend to be anxious and will not run the maze well otherwise. Prior to each testing day, the mice were adapted to the room with lights on for 15 min. For each session, all arms were baited with 0.1% saccharine solution and the animals were permitted to enter all arms until the eight rewards had been consumed. The number of errors, i.e., entries into previously visited arms and time to complete each session were recorded. An individual that was blinded to the status of the animal’s treatment performed the behavioral testing.

### Histology

Following behavioral testing, the mice were anesthetized with sodium pentobarbital (150 mg/kg, i.p.), perfused transaortically with 0.1 M PBS, pH 7.4. The brains were immediately removed and processed. The right hemisphere was immersion-fixed overnight in periodate-lysine-paraformaldehyde. Serial coronal brain sections (40 μm) were cut (approximately 40 sections in total), of which 10 were stained for histological and immunohistochemical analysis with: (i) a mixture of anti-Aβ mAbs 6E10 (Biosource, Camarillo, CA, USA) and 4G8 (Biosource); (ii) polyclonal anti-glial fibrillary acidic protein (anti-GFAP) antibody; or (iii) anti-Iba-1 antibody. 6E10 and 4G8 are mAbs that recognize Aβ and stain both pre-amyloid and Aβ plaques. The staining was performed with a combination of the antibodies, as each labels a portion of amyloid plaques, while the combination labels all plaques. GFAP is a component of the glial intermediate filaments that form part of the cytoskeleton in astrocytes and is often employed as a marker of astrocyte activation. Iba-1 is a commonly used marker for microglial activation at both early and later stages of plaque development. All procedures were performed by an individual blinded to the experimental study and conducted on free-floating sections, in principle as previously described (Boutajangout et al., 2009, 2017; Scholtzova et al., 2009, 2014, 2017; Liu et al., 2014). Briefly, sections were incubated with primary mouse monoclonal anti-Aβ antibodies 6E10 and 4G8 (Covance Research Products Inc., Denver, PA, USA) at 1:1,000 dilution for 3 h, secondary biotinylated mouse anti-mouse IgG antibody for 1 h at 1:2,000 dilution, and subsequent avidin-peroxidase complex for 30 min at the same dilution. The sections were thereafter reacted in 3, 3-diaminobenzidine tetrahydrochloride with nickel ammonium sulfate (Mallinckrodt, Paris, KY, USA) color intensification solution. GFAP immunostaining was performed by incubation with primary polyclonal anti-GFAP (Dako Inc., Carpinteria, CA, USA) at a 1:1,000 diluent composed of 0.3% Triton X-100, 0.1% sodium azide, 0.01% bacitracin, 1% bovine serum albumin (BSA), and 10% normal goat serum in PBS for 3 h, followed by secondary biotinylated goat anti-rabbit antibody (Vector Laboratories Inc., Burlingame, CA, USA) for 1 h at 1:1,000 dilution. Iba-1 immunohistochemistry was performed similarly to that for GFAP staining with the exception that a secondary goat anti-rat antibody was used (1:1,000, Vector Laboratories Inc., Burlingame, CA, USA). Equally spaced sections were mounted and stained in a solution containing 10% potassium ferrocyanide and 20% hydrochloric acid for 45 min. Stained sections were examined for each mouse and the average number of iron positive profiles per section was calculated.

Perl’s iron stain was performed on another set of sections to detect cerebral microhemorrhages/bleeding. Sections were stained in a solution containing 5% potassium ferrocyanide and 10% hydrochloric acid for 30 min. This method has been used in previous reports that showed that passive immunization against Aβ increased the frequency of microhemorrhages in AD model mice (Pfeifer L. A. et al., 2002; Wilcock et al., 2004; Racke et al., 2005). Diamino benzidine intensification of the iron staining, which is useful for detecting low levels of iron in Aβ plaques, did not appear to improve sensitivity for detecting the microhemorrhages and was therefore not employed.

### Image Analysis—Quantification of Amyloid Burden

Amyloid burden was quantified by a Bioquant stereology image analysis system (BIOQUANT Image Analysis Corporation, Nashville, TN, USA), using a random unbiased sampling scheme. Approximately 10 cortical and hippocampal sections, respectively, were analyzed per animal. Total Aβ burden (defined as the percentage of test area occupied by Aβ immunoreactivity) was quantified on coronal plane sections stained with the mixture of anti-Aβ antibodies 6E10/4G8 as previously described (Scholtzova et al., 2017; Liu et al., 2017; Goñi et al., 2018; Herline et al., 2018a). Intensification with nickel ammonium sulfate resulted in Aβ deposits being labeled black with minimal background staining that facilitated thresholding. Reactive astrocytosis was rated on a scale of 0–4. The rating was based on a semi-quantitative analysis of the extent of GFAP immunoreactivity (number of GFAP immunoreactive cells and complexity of astrocytic branching), as previously published (Goñi et al., 2013). The assessment of the Iba-1 immunostained sections was based on a semiquantitative analysis of the extent of microgliosis (0, none; 1, a few resting microglia; 2, moderate number; 3, numerous ramified/phagocytic microglia; 4, high number of microglia) in increments of 0.5, as previously reported (Scholtzova et al., 2009; Liu et al., 2014). Sections were analyzed per animal by an investigator who was blinded to the treatment status of the mice.

### Statistical Analysis

Data from the accelerating rotor rod and locomotor test were analyzed by one-way ANOVA. The data collected from the radial arm maze test was analyzed by two-way measures ANOVA followed by a Neuman-Leuls *post hoc* test. Differences between groups in total amyloid burden, activated microglia (Iba-1), GFAP astrogliosis, brain microhemorrhages, were analyzed using a Student’s unpaired two-tailed *t*-test or one-tailed *t*-test. All statistical tests were performed using Prism 6.0 (Graphpad, San Diego, CA, USA).

### CSF Bioavailability in Naïve Rats

Plasma and cerebrospinal fluid (CSF) profiles of the Aβ-binding affibody molecule were assessed to determine the CSF bioavailability in naïve rats. Rat care and experimental procedures were granted by the regional animal experimental ethics committee in Stockholm (North, N81/14). The rats were kept at Adlego Biomedical AB and maintained on a 12 h light/dark cycle. Sixteen male Sprague-Dawley rats were divided into two study groups that received intravenous injections of equimolar amounts (119 nmol/kg) of either Z_SYM73_-ABD or an Aβ-binding mAb (U-Protein Express, Utrecht, Netherlands) for comparison (both at 5 mL/kg). Prior to sampling of blood and CSF (at times 0, 3, 24, and 120 h), the animals were anesthetized with Ketaminol (75 mg/kg) + Rompun (10 mg/kg). CSF was sampled from *Cisterna magna* under anesthesia by puncturing the membrane between the occipital bone and the C1 cervical vertebrae using a needle. The membrane was exposed by opening the skin that covers the occipital bone with a minimal incision and the underlying muscles were separated by tweezers. Serum samples were prepared from blood by centrifugation. During the treatment, the rats were weighed and examined for general health indicators at each day of blood sampling. CSF and plasma samples were analyzed for Z_SYM73_-ABD or the Aβ-binding mAb by ELISA. Briefly, a mouse monoclonal anti-affibody was coated at 2 μg/mL in a 96 well PS microplate (Costar) overnight. Wells were washed in PBST (PBS with 0.05% Tween 20), and subsequently blocked in BlockerCasein-PBS (BCP, ThermoScientific) for 1.5 h at RT. Samples (at 20,000 pg/mL) were added to the wells, followed by addition of serum diluted 100- or 1,000-fold. For detection, primary antibody polyclonal rabbit anti-ABD antibodies were added to each well at 3 μg/mL, and incubated for 1.5 h at RT. Secondary reagent Jackson anti-rabbit IgG-HRP was added at 100 ng/mL for 1 h at RT. To develop the ELISA, ImmunoPure TMB was added to each well according to the manufacturer’s recommendations.

## Results

### Production of Z_SYM73_-ABD and (Z_Taq_)_2_-ABD

**Figure 1:**
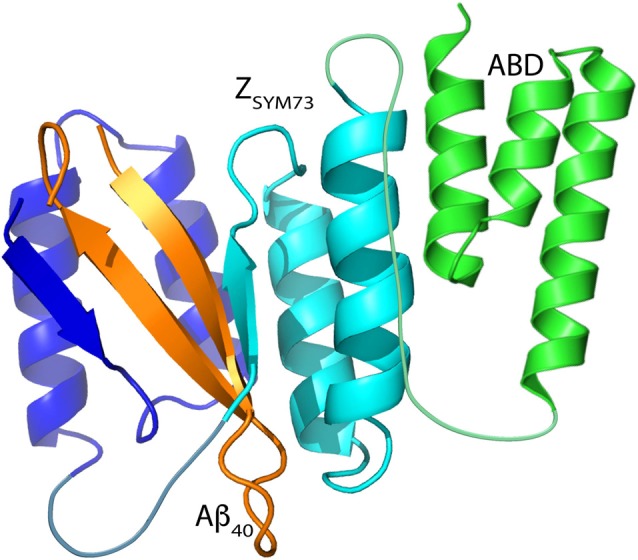
Schematic representation of Z_SYM73_-albumin-binding domain (ABD) in complex with amyloid β_1–40_ (Aβ)_1–40_. Figure adapted from Protein Data Bank entry 2OTK (Z_SYM73_:Aβ_1–40_) and Protein Data Bank entry 1GJS (ABD). The affibody subunits are illustrated in blue and cyan, the β-hairpin forming Aβ peptide is illustrated in orange, and the ABD is illustrated in green.

The therapeutic candidate was designed as a bispecific fusion protein, consisting of a genetic linkage between the high-affinity Aβ-capturing Z_SYM73_ affibody molecule and an ABD (Figure 1; Lindberg et al., 2015). In this format, Z_SYM73_-ABD (16.8 kDa) would be able to capture soluble Aβ peptides while simultaneously binding and circulating with HSA and thus extending the *in vivo* half-life of the protein (Andersen et al., 2011; Frejd, 2012). The ABD-moiety used in this study was a deimmunized variant with an engineered femtomolar affinity for HSA. The ABD also binds to serum albumin from other species, including murine serum albumin (MSA) to allow relevant preclinical evaluations (Jonsson et al., 2008; Frejd, 2012). A size-matched dimeric (Z_Taq_)_2_-ABD affibody molecule binding to DNA polymerase from *Thermus aquaticus*, was included as a negative control protein. The two fusion proteins were produced in *E. coli* and purified by affinity chromatography, reverse phase chromatography and size exclusion chromatography. Endotoxin content was analyzed and found to be below 0.15 EU/mL. In addition to this, a hexahistidine-tagged Z_SYM73_ protein was also produced and purified, as described elsewhere (Lindberg et al., 2015).

### Characterization of the Z_SYM73_ Affibody Fusion Protein

The purified proteins were analyzed by SDS-PAGE and MALDI-MS. The molecular masses were measured to 16780.3 Da for Z_SYM73_-ABD and 18662.8 Da for (Z_Taq_)_2_-ABD (Supplementary Figure S1A,B), which was in agreement with the expected theoretical values of 16782 Da and 18672 Da, respectively. The secondary structure content and refolding of Z_SYM73_-ABD was investigated using CD spectroscopy. CD spectra were recorded before and after the variable temperature measurement and showed almost complete overlap, suggesting reversible folding after heat-induced denaturation (Supplementary Figure S1C). The equilibrium dissociation constants and kinetic association rate constants of both Z_SYM73_-ABD and Z_SYM73_-His_6_ were investigated using a KinExA. The results demonstrated that the hexahistidine-fused Z_SYM73_ binds Aβ_40_ with a K_D_ ~12 pM and Aβ_42_ with a K_D_ ~21 pM (Table 1), corresponding to approximately 28-fold higher affinity than previously reported using a surface-based biosensor assay (Lindberg et al., 2015). Z_SYM73_-ABD demonstrated affinities of ~60 pM to both Aβ_40_ and Aβ_42_ while simultaneously binding HSA with an equilibrium dissociation constant of ~50 pM (Table 1). The ability of Z_SYM73_-ABD to bind Aβ from a plasma sample at 37°C was investigated over time in an ELISA (Supplementary Figure S2). The results showed that Z_SYM73_-ABD retained its binding activity for at least 3 days in human plasma, compared to a non-treated sample.

**Table 1:** Equilibrium dissociation constants (K_D_), association rate constants (k_a_), and dissociation rate constants (k_d_) for the amyloid β (Aβ)-binding Affibody molecules, determined by KinExA.

Molecule	Measured interaction	K_D_ (pM, the best KinExA fit and 95% CI^a^)	k_a_ (M^−1^s^−1^, the best KinExA fit and 95% CI^a^)	k_d_ (s^−1^)
Z_SYM73_-His6	Aβ_40_	12.1 (9.02–15.9)	7.45 (6.97–7.93) × 10^5^	9.01 × 10^−6^
Z_ SYM73_-His6	Aβ_42_	21.3 (15.6–28.4)	9.99 (8.29–11.8) × 10^5^	2.13 × 10^−5^
Z_ SYM73_-ABD	Aβ_40_	68.6 (39.8–106)	4.50 (4.25–4.74) × 10^5^	3.07 × 10^−5^
Z_ SYM73_-ABD	Aβ_42_	61.4 (40.1–88)	1.92 (1.8–2.04) × 10^6^	1.18 × 10^−4^
Z_SYM73_-ABD	HSA	53.7 (20–98.2)	5.08 (4.58–6.62) × 10^5^	2.73 × 10^−5^

*^a^The 95% confidence interval of the KinExA fit is shown in parentheses*.

### Preventive Treatment of 2xTg Mice and Safety Assessment

**Figure 2:**
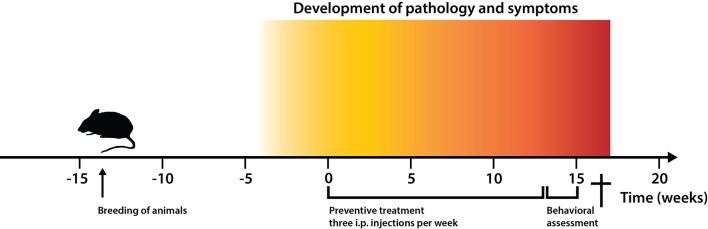
Experimental timeline for the treatment strategy of 2xTg Alzheimer’s disease (AD) mice. Timeline showing time points for: (i) breeding of animals with double transgenes amyloid precursor protein (APP_K670N/M671L_ and PS1_M146V_) for vascular amyloid deposition; (ii) pathology development (3 $\frac{1}{2}$ months); (iii) start and duration of preventive treatment with the Aβ-binding Z_SYM73_-ABD affibody molecule and control (Z_Taq_)_2_-ABD; and (iv) behavioral assessment of animals.

Mice with APP/PS1 (APP_K670N/M671L_ and PS1_M146V_) transgenes develop early and progressive vascular amyloid plaque pathology and were thus used to examine the preventive efficacy of the high-affinity Z_SYM73_-ABD candidate on the development of Aβ pathology. A scheme for the treatment strategy is outlined in Figure 2. At the onset of pathology (at approximately 3 $\frac{1}{2}$ months of age) and continuing for 13 weeks, each of 10 mice received three intraperitoneal (i.p.) injections of 100 μg Z_SYM73_-ABD (corresponding to a plasma concentration of ~1.5 μM that is estimated to fall to ~0.2 μM after 3 days) or the negative control protein (Z_Taq_)_2_-ABD per week. This amount was calculated to yield an equal or somewhat better exposure to that of concurrent mAbs when evaluated in mouse models. No differences in general health measures or any treatment-induced side-effects were observed across the experimental groups after the treatment (data not shown), indicating that the affibody fusion proteins were well tolerated and non-toxic. No antibodies that were reactive with Z_SYM73_-ABD could be detected after 39 i.p. injections (data not shown). These results correlate with results from a precursor of Z_SYM73_ that was previously evaluated as an ABD-fusion in rats, showing that no antibodies were detected to the fusion protein after 10 subcutaneous administrations over a period of 10 months (Fredrik Frejd, personal communication, International Publication Number WO 2005. /097202 A2).

### Circulatory Half-Life of Z_SYM73_-ABD

Plasma levels of Z_SYM73_-ABD were measured using ELISA prior to the first injection (T_0_), 24 h after the 39th injection (T_1_), then 7 (T_2_) and 14 (T_3_) days after the 39th injection. Based on this, the circulatory half-life of Z_SYM73_-ABD was estimated to approximately 35 h, which is in accordance with previous studies on ABD-fused affibody molecules in mice (Tolmachev et al., 2007; Supplementary Figure S3).

### Behavioral Studies

**Figure 3:**
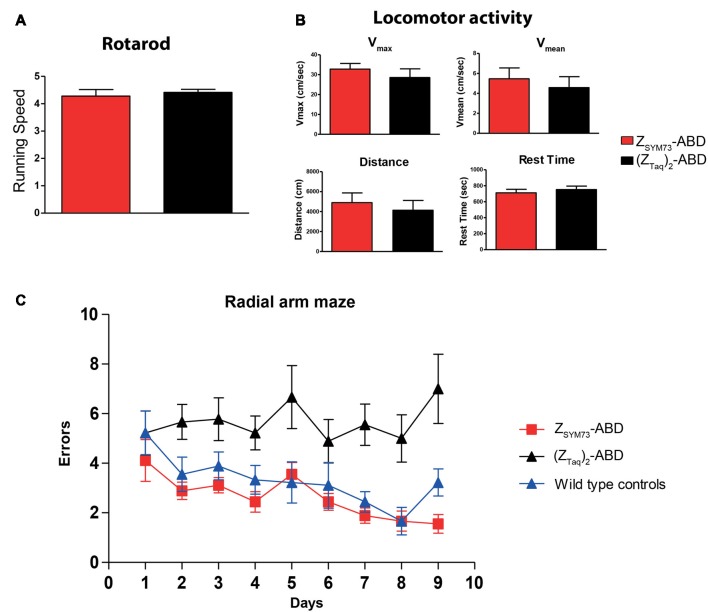
Cognitive testing and locomotor assessment of the affibody-administered APP/PS1 mice. **(A,B)** Assessment of sensorimotor abilities in 2xTg mice administered with the Z_SYM73_-ABD affibody molecule (red) or control (Z_Taq_)_2_-ABD (black) in **(A)** rotarod and **(B)** open field locomotor tests in which distance traveled, resting time, maximum velocity and average speed of mice were tested. **(C)** Radial arm maze testing (working memory assessment) of the Z_SYM73_-ABD (red) and (Z_Taq_)_2_-ABD (black) treated 2xTg mice. A group of wild type healthy control mice was included for comparison (blue). Animals were allowed to navigate the maze once per day for nine consecutive days. The number of errors that the mice performed on each testing day is plotted vs. the days of testing (*p* < 0.0001, by two-way ANOVA for treatment effect).

After the treatment period, both groups were subjected to behavioral testing. To verify that the results from the cognitive tests were not confounded by differences in sensorimotor abilities between the animals, sensorimotor testing was first conducted. No statistical differences were observed between the groups in rotarod (Figure 3A) or locomotor activity in terms of distance traveled, maximum speed, mean velocity and rest time (Figure 3B). The two groups, and an age-matched non-transgenic control group were subsequently tested in an eight-arm radial maze. The Z_SYM73_-ABD treated animals showed significant cognitive rescue as they navigated the maze with fewer errors than mice treated with the (Z_Taq_)_2_-ABD control protein (*p* < 0.0001 ANOVA two-way repeated measures for treatment effect). In addition, their performance was equal to that of the age-matched wild-type control group (Figure 3C), demonstrating that the Z_SYM73_-ABD treatment had a significant beneficial effect on the cognitive functions in this mouse model.

### Histological Quantification of Amyloid Burden

**Figure 4:**
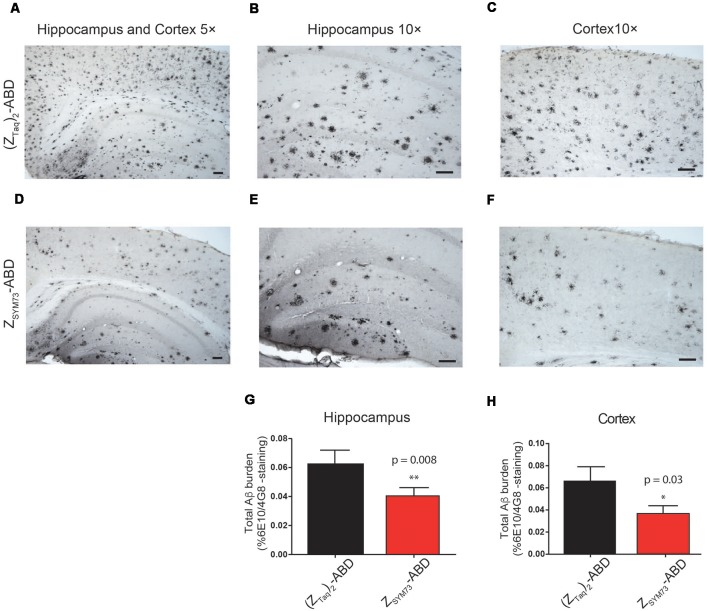
Histological evaluation of total amyloid brain burden. Representative immunohistochemical images and quantitative serological analysis of total amyloid burden using Aβ-binding antibodies 6E10/4G8 on brain sections from Z_SYM73_-ABD (red) or (Z_Taq_)_2_-ABD (black) control-treated mice, at 5× magnification **(A,D)**. Amyloid reduction in cortical (46% reduction, *p = 0.03, one-tailed *t*-test; **C,F,H**) and hippocampal (37% reduction, **p = 0.008, one-tailed *t*-test; **B,E,G**) brain sections at 10× magnification, scale bar (100 μm).

The preventive effect of Z_SYM73_-ABD was further assessed in terms of total amounts of amyloid burden in the regions of cortex and hippocampus (Figures 4A–H). The mice were sacrificed and their brains were processed for histology. Quantification of stained brain slices showed significantly lower amyloid burden (percentage area occupied by 4G8/6E10 immunoreactivity) for the Z_SYM73_-ABD treated APP/PS1 Tg mice compared to the control group in both the cortex (46% lower, *p* = 0.03 one-tailed *t*-test, Figures 4C,F,H) and hippocampus (37% lower, *p* = 0.008 one-tailed *t*-test, Figures 4B,E,G).

### Neuroinflammatory Response and Brain Microhemorrhages After Treatment

**Figure 5:**
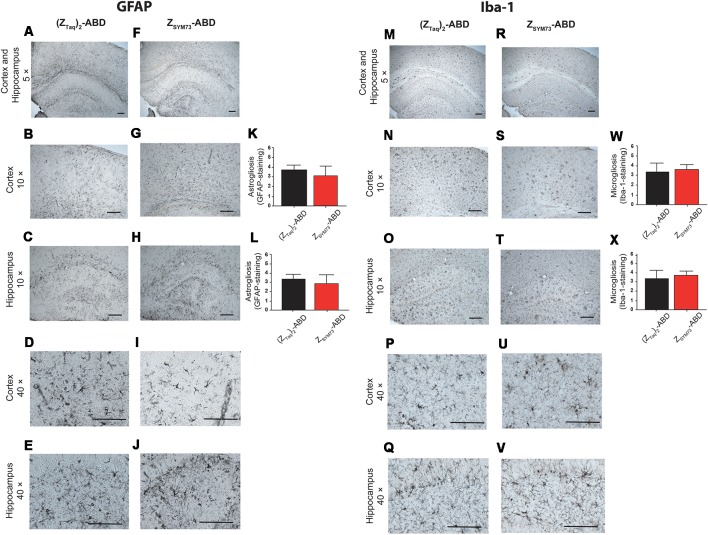
Analysis of glial fibrillary acidic protein (GFAP) and Iba-1 immunoreactivity post administration. Histological observations and semiquantitative rating of astrocyte activation by GFAP **(A,F)** and microglial cell activation by Iba-1 **(M,R)** reactivity on brain sections from Z_SYM73_-ABD (red) or (Z_Taq_)_2_-ABD (black) control-treated mice, at 5× magnification. GFAP-reactivity on cortical **(B,G,K)** and hippocampal **(C,H,L)** sections (*p* = 0.11 and *p* = 0.06, respectively); one-tailed *t*-test at 10× magnification, and 40× magnification **(D–J)**, respectively. Iba-1 microglia cell reactivity on brain sections at 5× magnification. Iba-1 reactivity on cortical (*p* = 0.25, one-tailed *t-*test; **N,S,W**) and hippocampal (*p* = 0.15, one-tailed *t*-test; **O,T,X**) sections at 10× magnification, and at 40x magnifications **(P–V)**, respectively. Scale bar (100 μm).

**Figure 6:**
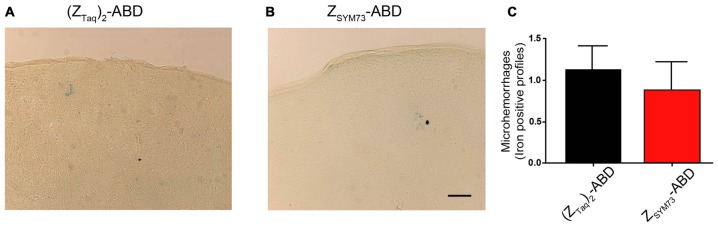
Semi-quantitative analysis of cerebral microhemorrhages. Representative brain sections demonstrating the degree of cerebral microhemorrhages in from (ZTaq)2-ABD **(A)** control or ZSYM73-ABD **(B)** treated mice by staining with Perl’s stain for ferric iron in hemosiderin. Semi-quantitation of iron positive profiles is shown in **(C)** (*p* = 0.29, one-tailed *t*-test). Scale bar (50 μm).

To evaluate the effect of the Z_SYM73_-ABD treatment on brain inflammation, serial sections were stained with anti-GFAP (Figures 5A–L) or anti-Iba-1 antibodies (Figures 5M–X), reactive to astrocytes and both active and resting microglia, respectively. Semi-quantitative analysis of GFAP immunoreactive cells and complexity of astrocytic branching showed no significant difference between treated and control groups in either the cortex (Figures 5B,G) or hippocampus (Figures 5C,H; *p* = 0.11 and *p* = 0.06, respectively; one-tailed *t*-test). Furthermore, administration of Z_SYM73_-ABD did not alter the microglia Iba-1 immunoreactivity in the cortex (Figures 5N,S) or hippocampus (Figures 5O,T) in the mice. Cerebral microhemorrhages were analyzed in brain sections stained with Perl’s stain and using semi-quantitative analysis. No significant differences between the Z_SYM73_-ABD treated animals and controls were observed in the amount of detected iron-positive profiles per brain section (*p* = 0.29; one-tailed *t*-test; Figures 6A–C). Taken together, this indicates that treatment with the Aβ-sequestering affibody did not result in neuroinflammatory responses or brain microhemorrhages.

### CSF Bioavailability in Naïve Rats

The potential site of action for affibody-mediated Aβ-sequestering was next investigated in a small pilot study. The uptake of Z_SYM73_-ABD to the CSF after administration was quantified in naïve rats, as CSF is difficult to extract from mice. Briefly, rats were administered with molar equivalents of either Z_SYM73_-ABD or an Aβ-binding mAb (U-Protein Express, Utrecht, Netherlands) as a control. CSF and plasma samples were extracted 0, 3, 24, and 120 h after the treatment, and the concentration measured using ELISA. The bioavailability in the CSF was determined to 0.13% and 0.12% for Z_SYM73_-ABD and the Aβ-binding mAb, respectively (Supplementary Figure S4), indicating that only a small fraction of the affibody molecule passes into the CSF. Based on this, it can be speculated that the preventive effect that was observed in mice was partly due to the sequestering of Aβ in blood as a complement to acting within the brain. However, further investigations are needed to determine the mode and site of action in detail.

## Discussion

We present a novel approach to prevent the development of Aβ-related pathology, using the Aβ-sequestering affibody molecule Z_SYM73._ The affibody molecule was previously engineered to sequester the aggregation-prone residues of monomeric Aβ by encapsulating the peptide in a tunnel-like cavity and thus preventing the peptide from forming neurotoxic aggregates.

To test the therapeutic potential of the Z_SYM73_ *in vivo*, the therapeutic candidate Z_SYM73_-ABD was first produced and characterized in terms of Aβ-binding activity and affinity in solution. In solution, the affibody molecule bound Aβ peptides with a 60 pM affinity while simultaneously binding to HSA *via* the ABD (also with picomolar affinity; Lindberg et al., 2015).

We show that Z_SYM73_-ABD can prevent the development and progression of Aβ pathology in APP/PS1 double transgenic mice with an early onset of pathology. Following three i.p. injections of 100 μg Z_SYM73_-ABD per week for 13 weeks, the treated animals exhibited significant cognitive rescue compared to mice treated with a size-matched (Z_Taq_)_2_-ABD control protein. The improved cognitive function was associated with a marked reduction in Aβ pathology. In addition, no affibody-specific antibody responses or immune-related side-effects were observed following the repeated administrations of the affibody protein. It has been speculated that Fc-activated immune cells can cause local inflammatory responses in the form of microhemorrhages (Pfeifer M. et al., 2002). However, affibody molecules lack the Fc domain of mAbs, and no such inflammatory responses were observed after the Z_SYM73_-ABD treatment.

It has been speculated that the small size of Z_SYM73_ (approximately 10-fold smaller than IgG) could possibly result in more efficient uptake in CSF compared to antibodies upon peripheral administration. To assess this, we investigated the CSF-uptake upon i.v. administration of Z_SYM73_-ABD or an Aβ-binding mAb in naïve rats. The bioavailability in the CSF was determined to 0.13% for Z_SYM73_-ABD and 0.12% for the Aβ-binding mAb, thus indicating a similar brain-uptake for both proteins. These results suggest that the size difference of the proteins had no dramatic influence on the passage over BBB, which leads us to further speculate whether the observed therapeutic effect in mice is a consequence of Aβ sequestration within the brain or through a peripheral sink effect (DeMattos et al., 2001; Citron, 2010). An important advantage for a significantly smaller protein drug is that a much higher molar dose can be administered in the same volume, which potentially could allow for subcutaneous injections and thus omit the demand for administration at an infusion center. Further studies are needed to elucidate the site of action. In future studies, it would be interesting to explore if antibody-based transferrin receptor-mediated transcytosis (Hultqvist et al., 2017) could improve BBB transfer of Z_SYM73_-ABD, and thus make it even more efficient in preventing the development of AD. mAbs to proteins such as α-synuclein and tau that are associated with other neurodegenerative diseases are also showing promise therapeutically (Herline et al., 2018a; Jankovic et al., 2018). Our affibody approach can also be designed to target these other pathology-associated proteins, with the same advantages as discussed above. Hence, the findings discussed here, have potential applicability to multiple neurodegenerative disorders.

To our knowledge, this is the first *in vivo* investigation of a systemically delivered scaffold protein targeting monomeric Aβ, which demonstrates a therapeutic potential for treatment of AD. With the recent set backs in clinical trials using Aβ-targeting mAbs, the path forward is indeed challenging (Herline et al., 2018b; Schott et al., 2019). However, this study supports further evaluations on non-immunoglobulin-based biopharmaceutical candidates for AD to assess differences and opportunities in the mode of action, biodistribution, toxicity profile et cetera compared to antibodies. Moreover, given that AD patients would most likely require long preventive treatment regimens for a relatively large part of the population, the small size of affibody molecules should be important, allowing for subcutaneous injections without assistance from an intravenous infusion center.

## Data Availability

All datasets generated for this study are included in the manuscript and/or the Supplementary Files.

## Author Contributions

AB: overall responsibility for the mouse studies, including breeding of mice, injections, behavioral tasks, histological analysis and plasma studies, involved in planning of the study, evaluation of the results, writing the manuscript. HL: generation of the Z_SYM73_-ABD and the control protein, production, purification and characterization, took part in the mouse studies performed at NYU, involved in evaluating the results, writing the manuscript. AA: performed a majority of the brain processing and some of the quantification of the histology. IA: performed the brain processing and some of the quantification of the histology. AP: assisted in the mice studies by quantitation of the findings on brain sections. RB: assisted in the mice studies and preparing reagents. IH-G: performed the CSF bioavailability study. EG: responsible for the KinExA study, writing part of the manuscript. FF: as an expert in the affibody technology, overall responsibility for the production and characterization of the affibody-fusion proteins. TH: as an expert in protein structures, responsibility for drug design and structural considerations. JL: as an expert in protein engineering, the design of the candidate drug, evaluation of the results, writing the manuscript. SS: involved in planning of the study, evaluation of the results, writing the manuscript. TW: overall planning of the study, evaluation of the results, writing the manuscript.

## Conflict of Interest Statement

IH-G, EG, and FF are employees at Affibody AB. JL and SS are members of the technical advisory board at Affibody AB. The remaining authors declare that the research was conducted in the absence of any commercial or financial relationships that could be construed as a potential conflict of interest.
