# Supplementary material for: Affibody-Mediated Sequestration of Amyloid β Demonstrates Preventive Efficacy in a Transgenic Alzheimer’s Disease Mouse Model
Source: Front Aging Neurosci. 2019 Mar 22;11:64. doi: 10.3389/fnagi.2019.00064 (PMC6440316; doi:10.3389/fnagi.2019.00064)
Supplement: Supplementary file 1 [file Data_Sheet_1.PDF]

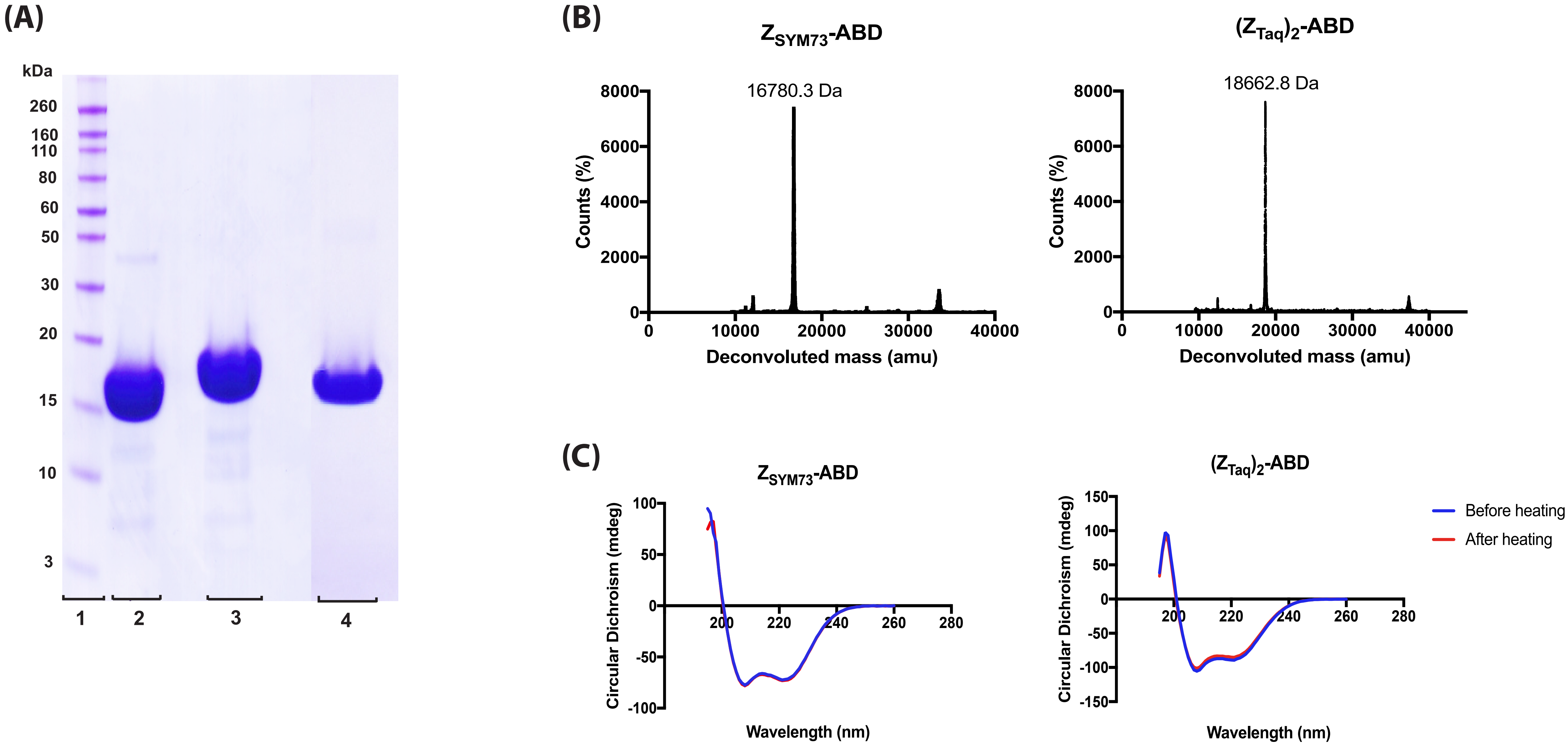

Supplementary figure 1 Characterization of Z_{SYM73}-ABD and $(Z_{Taq})_2$-ABD proteins. (A) SDS-PAGE analysis of proteins after purification. Lane 1, Novex sharp protein standard; lane 2, Non-reduced Z_{SYM73}-ABD (16.8 kDa); lane 3, reduced Z_{SYM73}-ABD, lane 4, $(Z_{Taq})_2$-ABD (18.7 kDa). **(B)** Mass spectrometry analysis of the purified proteins. **(C)** Circular dichroism (CD) spectra of Z_{SYM73}-ABD and $(Z_{Taq})_2$-ABD before and after heating to 90°C, at wavelengths ranging from 195 nm to 250 nm at 20°C.
