# Supplementary material for: Affibody-Mediated Sequestration of Amyloid β Demonstrates Preventive Efficacy in a Transgenic Alzheimer’s Disease Mouse Model
Source: Front Aging Neurosci. 2019 Mar 22;11:64. doi: 10.3389/fnagi.2019.00064 (PMC6440316; doi:10.3389/fnagi.2019.00064)
Supplement: Supplementary file 2 [file Data_Sheet_2.PDF]

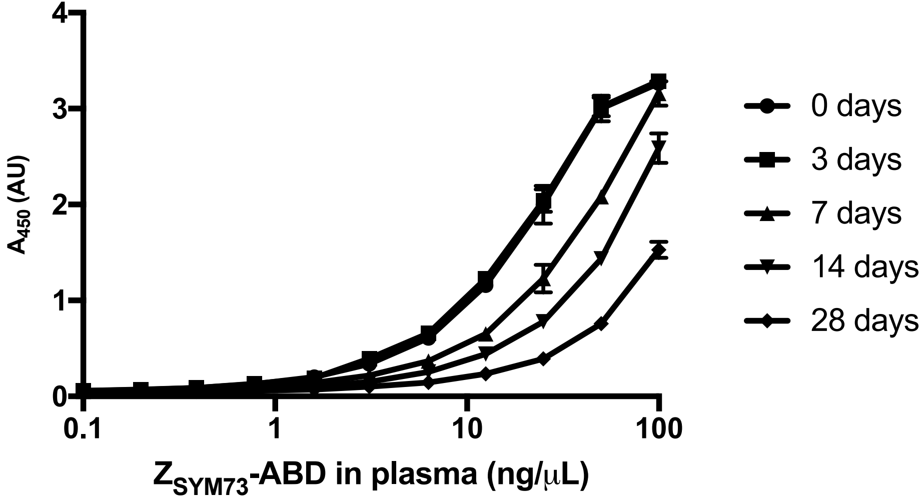

Supplementary figure 2 ELISA analysis of Z_{SYM73}-ABD stability in serum. Z_{SYM73}-ABD incubated in human plasma at 37°C for 0, 3, 7, 14 and 28 days, followed by analysis of retained binding to $A\beta_{40}$ coated in wells. Z_{SYM73}-ABD was detected using a HRP-conjugated mouse anti-affibody mAb followed by a one-step ultra-TMB substrate. Please note that some error bars are too small to be displayed in the image.
