# Supplementary material for: Affibody-Mediated Sequestration of Amyloid β Demonstrates Preventive Efficacy in a Transgenic Alzheimer’s Disease Mouse Model
Source: Front Aging Neurosci. 2019 Mar 22;11:64. doi: 10.3389/fnagi.2019.00064 (PMC6440316; doi:10.3389/fnagi.2019.00064)
Supplement: Supplementary file 3 [file Data_Sheet_3.PDF]

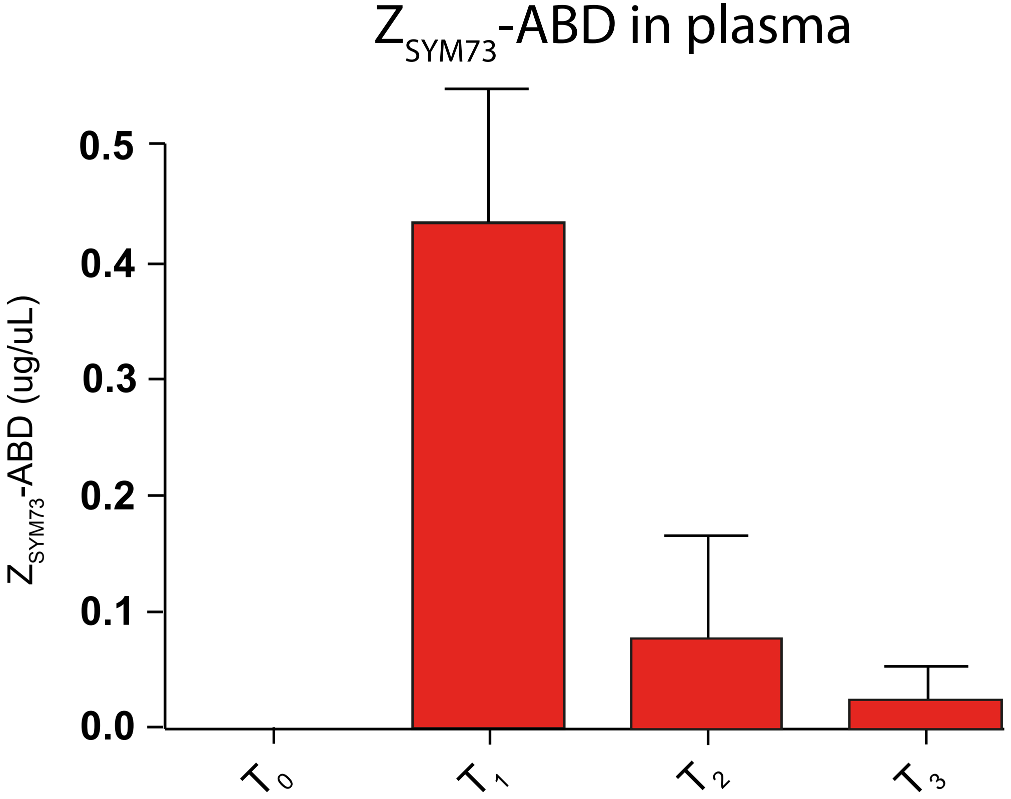

Supplementary figure 3 Plasma levels of ZSYM73-ABD in 2×Tg mice. ELISA was used to assess plasma levels of affibody after administration. Aβ peptide was coated at the bottom of 96-well microtiter wells, following detection of affibody molecules in diluted plasma samples using a goat α-affibody IgG. Bound anti-affibody antibodies were detected using biotinylated anti-goat IgG, followed by a Streptavidin HRP and tetramethyl benzidine (TMB) substrate. T0: prior to the first injection; T1: 24h after the 39th injection; T2: 7 days after the 39th injection, and T3: 14 days after the 39th injection.
