# Supplementary material for: Affibody-Mediated Sequestration of Amyloid β Demonstrates Preventive Efficacy in a Transgenic Alzheimer’s Disease Mouse Model
Source: Front Aging Neurosci. 2019 Mar 22;11:64. doi: 10.3389/fnagi.2019.00064 (PMC6440316; doi:10.3389/fnagi.2019.00064)
Supplement: Supplementary file 4 [file Data_Sheet_4.PDF]

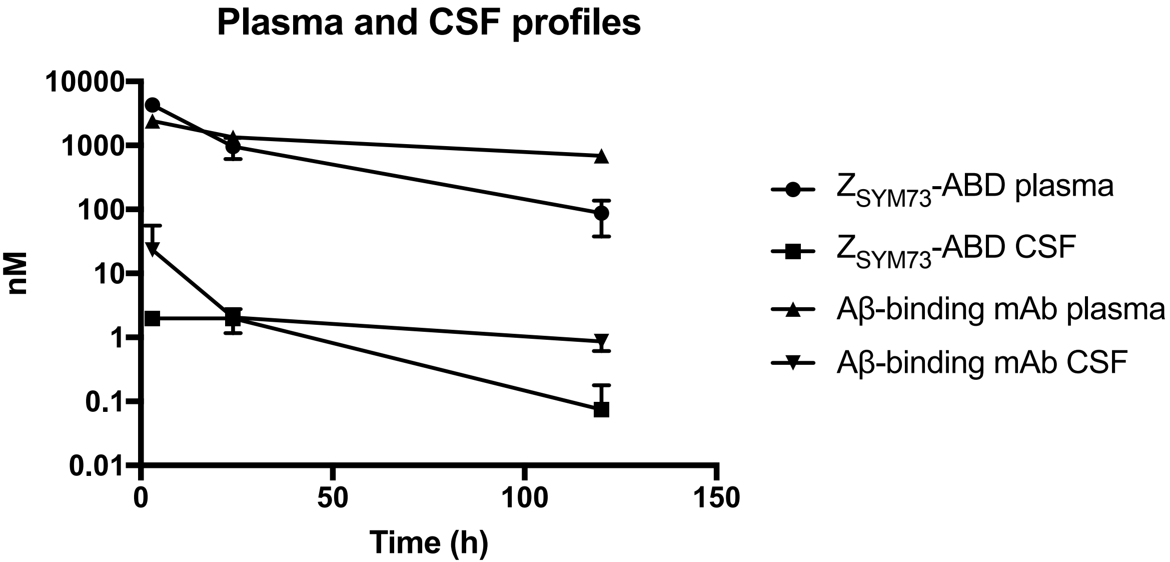

Bioavailability CSF

Z_{SYM73}-ABD 0.13%   Aβ-binding mAb 0.12%

Supplementary figure 4 Plasma and cerebrospinal fluid profiling of Z_{SYM73}-ABD in naïve rats. Molar equivalents of Z_{SYM73}-ABD or an Aβ-binding control mAb were administrated to naïve rats, followed by ELISA analysis of plasma and cerebrospinal fluid (CSF) content. Diluted samples were added to wells pre-coated with a mouse monoclonal anti-affibody, followed by detection with polyclonal rabbit anti-ABD094 antibodies and secondary reagent Jackson anti-rabbit IgG-HRP. Please note that some error bars are too small to be displayed in the image.
